# Species Accumulation Curves and Incidence-Based Species Richness Estimators to Appraise the Diversity of Cultivable Yeasts from Beech Forest Soils

**DOI:** 10.1371/journal.pone.0023671

**Published:** 2011-08-12

**Authors:** Andrey M. Yurkov, Martin Kemler, Dominik Begerow

**Affiliations:** 1 Geobotany, Department of Evolution and Biodiversity of Plants, Faculty of Biology and Biotechnology, Ruhr-Universität Bochum, Bochum, Germany; 2 Centre of Excellence in Tree Health Biotechnology, Forestry and Agricultural Biotechnology Institute (FABI), University of Pretoria, Pretoria, South Africa; University College London, United Kingdom

## Abstract

**Background:**

Yeast-like fungi inhabit soils throughout all climatic zones in a great abundance. While recent estimations predicted a plethora of prokaryotic taxa in one gram of soil, similar data are lacking for fungi, especially yeasts.

**Methodology/Principal Findings:**

We assessed the diversity of soil yeasts in different forests of central Germany using cultivation-based techniques with subsequent identification based on rDNA sequence data. Based on experiments using various pre-cultivation sample treatment and different cultivation media we obtained the highest number of yeasts by analysing mixed soil samples with a single nutrient-rich medium. Additionally, several species richness estimators were applied to incidence-based data of 165 samples. All of them predicted a similar range of yeast diversity, namely 14 to 16 species. Randomized species richness curves reached saturation in all applied estimators, thus indicating that the majority of species is detected after approximately 30 to 50 samples analysed.

**Conclusions/Significance:**

In this study we demonstrate that robust species identification as well as mathematical approaches are essential to reliably estimate the sampling effort needed to describe soil yeast communities. This approach has great potential for optimisation of cultivation techniques and allows high throughput analysis in the future.

## Introduction

Soils display a remarkable heterogeneity throughout all climate zones, thus providing a multitude of diverse habitats of different scales and properties. Many studies on the biological diversity associated with particular soil types suggest that soil, in general, may well be a megadiverse habitat dominated by invertebrates, prokaryotes and fungi [1]–[4]. Fungi living in soils can be divided in two functional groups: filamentous, multicellular fungi and unicellular, yeasts. Yeasts comprise a systematically artificial group of fungi, which includes members of various orders of both Asco- and Basidiomycota [5]–[6]. The knowledge of yeast species diversity has tremendously increased over the last 50 years with nearly 1500 described species by 2010 [7] and estimations of the total number of yeast species assuming up to to 15,000 species, respectively [8].

Soil yeasts are known from the Polar Regions to the tropics and in total up to 130 species were reported worldwide. However, evidence for a strong association with soil-related substrates is lacking for many of these species [6], [9]–[11] and despite a growing number of studies assessing soil biodiversity using culture-independent techniques [12]–[14], our knowledge of soil-inhabiting yeasts is mostly derived from cultivation-based approaches [6]. In general, diversity measures rely not only on cultivation success but also on sufficient sampling and reliable species recognition, e.g. [15]–[17]. Although identification methods based on ribosomal DNA sequencing gained more and more importance in the last decade [5]–[6], [18], most of the studies, which were conducted in forest soils, utilized a combination of morphological and physiological characters for species identification. This is however problematic, because several soil-related yeasts, e.g. *Cryptococcus albidus*, *Cr. humicola*, *Cr. laurentii*, are taxonomically complex and may in fact be difficult to distinguish [19]–[21]. Therefore, separation of closely related and morphologically similar cryptic species using molecular tools could considerably influence the assessment of the existing microbial diversity.

Yeast species richness studies in forest soils of the temperate zone that were based on conventional phenotypic methods reported 18 to 26 yeast species [22]–[24]. Surprisingly, the only currently available study attempting species identification on the basis of rDNA sequence data obtained similar species numbers (i.e., 19 and 24 from two different forest sites, respectively) [25]. As molecular techniques are more sensitive in detecting cryptic species this result might indicate some methodological problems (e.g. undersampling), which would lead to a species underestimation.

Because microbial diversity differs largely across habitats, no meaningful comparison of biodiversity assessments can be performed without understanding the ranges of alpha-diversity, i.e. species richness, in a substrate or biotope. We used soil yeast community data to address three basic questions, which have previously not been considered within a single study: (1) Does soil heterogeneity have an effect on the observed species richness? (2) Is cultivation success of different yeast species influenced by different cultivation media and thereby by differences in nutrient availability? (3) What is the expected diversity estimated in relation to sampling effort? Special attention was given to robust species identification by sequencing rDNA and subsequent phylogenetic analysis in order to address species recognition and its impact on estimations of the yeast species richness in soils.

## Materials and Methods

### Study site, soil sampling and pre-cultivation sample treatment

The study has been performed in the Biodiversity Exploratory Hainich-Dün (http://www.biodiversity-exploratories.de). An overview of the German Biodiversity Exploratories is given by Fischer at al. [26]. Samples were taken in beech (*Fagus sylvatica*) forests of two different management types. The near-natural unmanaged forest consisted of 100 years old beech stands sometimes mixed with *Fraxinus excelsior* and *Acer pseudoplatanus*. The managed forest type consisted of 40-year-old age-class planted forests, with the tree species being *Fagus sylvatica*, and beech selection cutting forest. Details on the study sites, their properties and the field permits are provided in Fischer et al. [26]. Soils were classified in the field according to FAO [27] and KA5 [28] guidelines as Luvisols (Parabraunerde).

**Figure 1:**
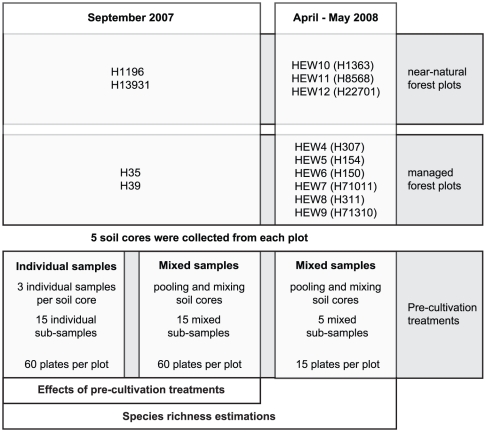
Scheme of the experimental design, sampling dates, analysed forest plots and applied pre-cultivation treatments.

During September 2007, soil cores (topsoil, A_h_ horizon) were collected using a steel ring (volume: 100 cm^3^), placed in sterile paper bags, transferred to the laboratory and kept at −20°C before analysis. Soil from one plot (five core samples) was mixed in equal proportion and sieved consequently through 10 mm, 2 mm, 1 mm and 0.065 mm meshes (Figure 1). Roots, stones and woody particles were thereby removed on every step. The soil was afterwards pooled again and five samples were taken to represent one plot. Each sample was inoculated in triplicates, i.e. 15 mixed soil sub-samples and 2 plates per sample, 60 plates in total. Individual samples taken from the same soil cores, were plated in the same way to have the same number of plates per plot in the end (Figure 1). From the 5 core samples taken on a single plot 3 sub-samples were randomly taken (15 sub-samples per plot) and plated on 2 plates each, 60 plates in total. Soils collected in April–May 2008 were studied as mixed samples. Details on soil sampling are given by Yurkov et al. [29].

### Isolation of cultures

Soil samples were placed in 50 ml plastic tubes, suspended (w/v) 1∶5, 1∶10, and 1∶20 in sterile water and shaken on an orbital shaker at 200 rpm for 1 hour. An aliquot of 0.15 ml was distributed on the surface of solid media. Glucose-yeast extract-peptone agar (GPYA) acidified with lactate (final pH 4.5), MYP agar [30] supplemented with 0.05% tetracycline, thymine–mineral–vitamin (TMV) agar [31] and modified Browns' nitrogen deficient media [32] supplemented with imidazol [33] and cycloheximid [34] were used for cultivation experiments. Plates with GPYA and MYP media were incubated at room temperature for 3 days and then at lower temperature (6–10°C) to prevent fast development of moulds. Plates with nitrogen deficient media were incubated at room temperature. All plates were checked after 7, 14 and 21 days of incubation. Colonies were differentiated into macro-morphological types using dissection microscopy, counted, and 1–2 representatives of every colony type per plate were transferred into pure culture.

### Identification of cultures

DNA was isolated from 3–4 days old cultures using a technique described by Hoffman and Winston [35], with slight modifications. DNA was precipitated with ethanol and then dissolved in 50 µl TE buffer containing RNAse (10 µg/ml). PCR-fingerprinting with minisatellite-specific oligonucleotides derived from the core sequence of bacteriophage M13 with the sequence given by Sampaio et al. [36] or microsatellite-specific oligonucleotides (GTG)_5_, (ATG)_5_ and (GAC)_5_ as single PCR primer [37] were used to group pure cultures. Strains showing identical electrophoretic profiles were considered as conspecific and only 1–2 representatives of them were chosen for further identification by sequencing of rDNA regions. DNA fragments were amplified by PCR using the primers ITS1f and NL4 [38]–[39]. Initial denaturation was performed at 96°C for 2 min, followed by 35 cycles of 20 s at 96°C, 50 s at 52°C and 1.5 min at 72°C, respectively. A final extension step of 7 min at 72°C was conducted.

PCR products were purified with the my-Budget Double Pure kit (Bio-Budget Tech., Germany) and sequenced on an ABI3130xl sequencer using the same primers as for PCR amplification. Chromatograms were checked and corrected with Sequencher 4.8–4.10 (Gene Codes Corp., USA). For species identification the obtained nucleotide sequences were compared with sequences deposited in the NCBI (www.ncbi.nih.gov) and CBS (www.cbs.knaw.nl) databases, respectively.

### Statistical data analyses

Yeast quantity was calculated as CFU (colony forming units) per gram of soil at natural humidity. The community structure was characterised by frequency of occurrence (incidence) of every observed species in the sample, which was calculated as the relative occurrence of the species to the total number of species in the sample. Statistical evaluations were performed with STATISTICA 8.0 (StatSoft, Inc., Tulsa, USA). The reliability of the different sampling assays was statistically tested by the comparison of results obtained from mixed and individual samples both in near-natural and managed forests using one-way analysis of variance. Only data that passed normality test were used for further analyses. Effects were considered to be statistically significant at the level p<0.05. Significant effects were additionally confirmed with Chi-square test. Species accumulation curves were calculated with EstimateS 8.0 using 50 randomizations, sampling without replacement and default settings for upper incidence limit for infrequent species [40]. As distinct yeast species could form colonies with similar morphology and, thus, make the separation to different types doubtful, we have used only presences/absences (incidence data) in our community matrix. The latter did not depend on the morphological differentiation but relied solely on molecular species identification. Four estimators of species richness were used: Chao 2 richness estimator [41], ICE incidence-based coverage estimator [42], Jackknife 1 first-order Jackknife richness estimator [43], and Bootstrap richness estimator [44]. Of the four species richness estimators, ICE distinguishes between frequent and infrequent species in analyses, Bootstrap does not differentiate the species frequency and the first-order Jackknife richness estimator additionally relies on the number of species only found once. Chao 2 estimator is distinct from the other species estimators as it is an incidence-based estimator of species richness, which relies on the number of unique units and duplicates (species found in only one and two sample units [45]). Species-area (i.e., species-plot) relationship was studied using the procedure described in Ugland et al. [46]. Because yeast communities sampled in September and April differed considerably, we did not use the full randomisation of the areas (plots). Instead, all analysed datasets contained samples from at least one plot sampled at September 2007 and at least one plot sampled in April 2008. For each dataset four species richness estimators were applied. Subsequently, average species richness values were calculated for each combination of samples comprising 2, 3, 4 and 5 plots. The number of samples allocated for the analyses of combinations of plots as well as the species richness values are provided in the Table S2.

## Results

### Effect of soil heterogeneity on species richness

The total yeast counts varied largely from 10^2^ to 10^4^ CFU/g. However, there was no significant difference related to the distinct sample treatments. 1.2×10^3^ CFU/g of yeasts were observed in mixed and 1.0×10^3^ CFU/g in individual samples (Figure S1).

**Figure 2:**
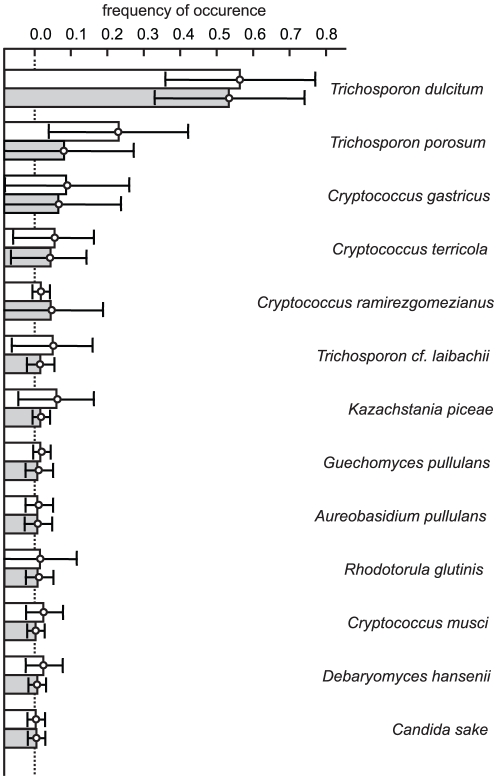
Frequency of appearance of yeasts in individual (white) and mixed (grey) soil samples. Whiskers correspond to confidential interval. Dotted line corresponds to zero frequency of appearance.

The observed community structures determined from the incidence of eleven shared species were highly similar both in mixed and individual samples (Figure 2 and Table S1). *Trichosporon dulcitum* was found to be the most frequent yeast species irrespective of pre-cultivation sample treatment and was observed in more than half of the analysed samples. *Cryptococcus terricola* and *T. porosum* were observed more frequently in individual samples whereas *Debaryomyces hansenii* and *Kazachstania piceae* were found more frequently in mixed samples. Even though average incidence values of these species differed 3–5 times between individual and mixed samples, confidential interval suggests these differences to be statistically insignificant (ANOVA, p = 0.053). The frequency of appearance of *Trichosporon porosum* was slightly higher in the individual samples. Nevertheless, in both cases it was the second dominating species (Figure 2 and Table S1).

### Observed species richness and effect of different media on species richness

We isolated 14 yeast species from near-natural beech forest soils and 12 from managed forest soils. In total we isolated 18 different species, from which 11 species were observed in both forest types (Table S1). The total species number (i.e. 18 species) was recovered by using GPYA and MYP media, and six species with TMV media (*Trichosporon dulcitum*, *Guehomyces pullulans*, *Kazachstania piceae*, *D. hansenii*, *Candida sake*, and *C. vartiovaarae*). Modified Brown's media was the most selective one and yielded isolates of only one species, *G. pullulans* (Table 1).

**Table 1:** Results of cultivation surveys: occurrence of yeasts in soils after different pre-cultivation treatments, in two distinct sampling periods, and with different cultivation media.

Effects:	Pre-cultivation treatments		Sampling date		Media
Species	Mixed	Individual	Sept-2007	Apr-2008	GPYA (G), TMV (T), Brown's agar (B)
*Aureobasidium pullulans*	×	×	×	n.o.	G
*Barnettozyma pratensis*	×	n.o.	n.o.	×	G
*B. vustinii*	×	n.o.	n.o.	×	G
*Candida kruisii*	×	×	×	×	G
*C. sake*	×	×	×	×	G, T
*C. vartiovaarae*	×	n.o.	n.o.	×	G, T
*Cryptococcus gastricus*	×	×	×	×	G
*Cr. musci*	×	×	×	n.o.	G
*Cr. ramirezgomezianus*	×	×	×	n.o.	G
*Cr. terricola*	×	×	×	×	G
*Debaryomyces hansenii*	×	×	×	×	G, T
*Guechomyces pullulans*	×	×	×	×	G, T, B
*Kazachstania piceae*	×	×	×	×	G, T
*Lindnera misumaiensis*	×	n.o.	n.o.	×	G
*Rhodotorula glutinis*	×	×	×	×	G
*Trichosporon dulcitum*	×	×	×	×	G, T
*Trichosporon* cf. *laibachii*	×	×	×	n.o.	G
*Trichosporon porosum*	×	×	×	×	G

Abbreviation used: GPYA, glucose-yeast-peptone agar; TMV, thimine-mineral-vitamine medium; n.o., not observed.

Dissimilarity in the yeast community composition was observed between soils collected in two different sampling dates (Table 1). Five yeasts, *Aureobasidium pullulans*, *Cryptococcus gastricus*, *Cr. musci*, *Cr. ramirezgomezianus*, and *Trichosporon* cf. *laibachii* were isolated only from the soil samples collected in September 2007, while *Barnettozyma pratensis*, *B. vustinii*, *C. vartiovaarae* and *Lindnera misumaiensis* were detected only in April–May 2008.

### Estimated species richness

**Figure 3:**
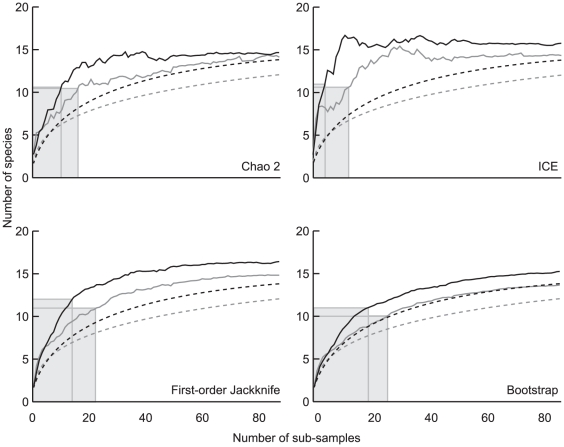
Estimator-based (solid line) and randomised (dashed line) species accumulation curves for near-natural (black) and managed (grey) beech forests obtained with incidence-based coverage (ICE), Chao 2, first-order Jackknife (Jack 1) and bootstrap richness estimators. Shadowed areas correspond to the 75% expected diversity cut-off.

Because yeast community composition and the frequency of occurrence varied widely among sub-samples (Figure 2), species richness estimators were applied to obtain reliable numbers of species to be expected with the conventional cultivation technique [17], [47]. When a total of N = 165 sub-samples were combined into two datasets, for the near-natural (N = 75) and managed (N = 90) forests, both of the species accumulation curves were close to saturation (Figure 3 and Table 2) showing that our study provides a reliable basis for yeast species richness assessment in a beech forest soils.

**Table 2:** Estimations of species richness obtained with different models (Mean and standard deviation).

Forest type	N of samples	Observed	ICE	Chao 2	Jackknife 1	Bootstrap
Natural	75	14	16.7±4.3	14.7±3.8	16.4±1.6	15.2±0.4
Managed	90	12	15.4±6.3	14.3±2.6	14.8±1.5	13.6±1.6
Total	165	18	18.6±0.4	19.0±2.3	20.0±1.4	19.0±0.02

Abbreviations used: ICE, incidence-based coverage estimator; Chao 2, Chao 2 richness estimator; Jackknife 1, first-order Jackknife richness estimator [38]–[41].

Species richness estimators calculated similar values for both forest types (Figure 3 and Table 2). Similarly to the obtained species accumulation curves, estimations were always higher, although not significantly, for natural than managed forests. Depending on the estimator used, means of estimated richness varied only in the range of about 2 species (Table 2). For managed and natural forests the ICE estimator with 15.4 and 16.7 yeasts respectively achieved the highest species richness. The lowest predicted values were 15.2 by Bootstrap in natural and 14.3 species by Chao 2 for managed forests. First order Jackknife estimator predicted relatively high species richness values together with low standard deviations in both forest types. Notably, the estimated species richness values did not differ significantly within a forest type (Table 2).

**Figure 4:**
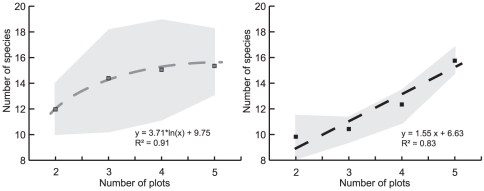
The average estimator based species accumulation curve for one to five plots of near-natural (black) and managed (grey) beech forests. Shadowed areas correspond to the standard deviation.

We estimated the species richness within an area to assess effects of community heterogeneity between the plots on the expected diversity. These estimations were performed for 2, 3, 4 and 5 plots, respectively (Table S2; Figure 4). On average, analysis of 3 managed plots predicts about 14 species to be found. Estimations performed with two additional plots result in 2 more species to be expected in soils under managed forests. Unlike the managed biotopes, near-natural beech forests displayed a contrasting trend, and the curve constructed using the estimations did not reach saturation after 3–4 plots but still increased continuously. In both forest types, the observed number of species followed the same trend as for the estimated species richness.

## Discussion

The main purpose of this study was to provide baseline data on the alpha-diversity of yeast fungi in soils under beech forests in central Germany. Although the conventional plating technique has been the preferred method for investigating yeast biodiversity for a long time [48]–[49], no reliable estimations of yeast species richness in soils were performed to this date. The discrepancy between observed and estimated yeast diversity [8], [50] is derived from different approaches of species detection and so far we do not know whether this is due to sampling biases or real limitations of the conventional plating technique. High-throughput dilution-to-extinction cultivation approaches improved the recovery of fungi from plant-related substrates [51]–[52], but has the same limitations as the applied plating technique. Due to the rise of massive parallel sequencing, culture-independent approaches of soil species diversity have received much attention in the last years, e.g. [3], [13]–[14]. However, yeasts as soil organisms have been often neglected in these studies and reliable data to validate the results from culture independent approaches are lacking. Cultivation from soil suspension enables analysis of soil samples 10–20 times larger than the ones used by culture-independent techniques, e.g. [3], [13]–[14]. In the present study we analysed a considerable amount of soil samples making a total of nearly 1 kg that renders our work the most comprehensive study utilized plating approach followed by molecular identification.

### Pre-cultivation sample treatments and scale-dependency of community structure

Analysis of individual and mixed samples was aimed to reveal effects of soil meso- and micro-heterogeneity on estimations of species richness. We hypothesised that patchiness of soil properties, like acidity, water activity, and availability of nitrogen and carbon sources could significantly affect soil yeast communities on the level of soil aggregates. Thus, homogenisations and pooling of soil samples should even the distribution of yeast populations when multiple samples are collected (and mixed) to represent the soil cover of a biotope. Total yeast counts in our study are comparable with the average numbers previously found for soil-related substrates in temperate climate zone [9], [22]–[23], and no significant effect of pre-cultivation sample treatment was observed (Figure 2). As determined from the incidence data, our investigation revealed that soil homogenisation does not significantly affect community structure. Differences in species occurrence were found to be more pronounced between natural and managed forests than between soil of individual and mixed samples (Table S1). Therefore, soil heterogeneity at the level of soil horizon and biotope seems to have no significant effects on the species richness of yeast fungi and the analysis of mixed soil samples provides reliable results and reduces time and costs. Additionally, the absence of pronounced effects of our pre-cultivation treatments suggests that spatial niche separation of soil yeast species might occur at a lower level of soil heterogeneity resembling bacterial communities distribution patterns [53].

### Effect of different media on observed species richness

Four different media, representing a gradient of nutrient availability were used in the cultivation experiment. Although various authors have suggested using selective media in order to obtain higher diversity [54]–[56], our results demonstrate that the use of just one nutrient-rich medium can reveal the majority of cultivable yeasts from soil. Specifically, both of the nutrient-rich media, GPYA and MYP, resulted in recovering the highest number of yeast species, namely isolation of all observed species. We did not observe any significant effect between MYP and GPYA media, which could be explained by the fact that most of the soil-inhabiting species are basidiomycetes that are known to have wide assimilation spectra [6]. By using two more oligotrophic and nitrogen-depleted media (TMV and Brown's agar), only up to six species were be isolated. Nitrogen deficient media were previously used for the isolation of the members of the genus *Lipomyces* because these soil yeasts have the rare ability among yeasts to utilize nitrogen from heterocyclic compounds, such as imidazole, pyrimidine, and pyrazine [33], [57]. These compounds were consequently included as a nitrogen source in selective media to isolate *Lipomyces* yeasts from soils [31], [58]. Members of the genus *Lipomyces* and their anamorphs *Myxozyma* (Lipomycetaceae, Saccharomycetales, Ascomycota) display a typical oligotrophic behaviour, slow growth on standard nutrient-rich media coupled with the ability to assimilate complex substrates. The ability of other yeasts to grow on medium containing heterocyclic compounds as the only source on nitrogen has been recorded only once [31]. We could not isolate any members of Lipomycetaceae with nitrogen-depleted media, but six other yeasts (Table S1). Thus, our results suggest that the ability to grow in the absence of external nitrogen sources and utilisation of heterocyclic compounds is more common among soil yeasts than has been assumed previously.

In addition to lower observed diversity, modified Browns' agar and TMV media seems not suitable for soil diversity studies in contrast to nutrient-rich media, because yeast colonies of different species on nitrogen-depleted media looked very similar, either extremely mucous or dimorphic (with developed substrate mycelium). After transfer from these media to GPYA they often split in several morphologically and taxonomically distinct cultures. Therefore, it is very difficult to make assumptions about the observed species abundance using these media. Additionally, we often observed strong development of moulds on our samples although TMV agar has been reported to prevent overgrowth of yeast colonies by filamentous fungi [31].

### Estimated species richness and community structure

Rarefaction curves were close to saturation and we applied species richness estimators to predict the number of species to be expected from the soils using the traditional plating technique (Figure 3). All of the applied richness estimators predicted 14 to 16 species per forest type, with a slightly higher (but not significant) diversity to be expected in natural forests. Estimator curves reached saturation starting from 30% to 50% of the analysed sub-samples depending on the estimator applied. In other words, the number of plates for the analysis can be significantly reduced. This result apparently reflects the soil yeast community structure, which is characterised by a few autochthonous species (e.g. *Trichosporon dulcitum*, *T. porosum*, *Cryptococcus terricola*) accounting for the majority of isolates, and a large number of minor or rare species. Interestingly, the same pattern, a species-poor yeast community with uneven distribution of species, is known for yeasts inhabiting floral nectar [17]. Unlike soils, nectar is nutrient-rich and is characterised by high cell densities. Nevertheless, in both cases environmental conditions strongly affect the yeast population and select towards a few specialist species. Nearly one third of 18 yeast species isolated in our study was rare (either unique species or duplicates) and could be detected in less than in 3% of the sub-samples (Table S1). In soils, a number of physico-chemical properties of which oligotrophy, water availability and complex organic acids are of the main importance [6] considerably shape the community and, as a result, a limited number of yeasts were common among the two forest management types (Table S1). Species-plot relationship reflects the importance of rare species for species richness estimations. In the studied areas, three remote forest sites separated by 20–25 km, the yeast community inhabiting soils of managed forests could be assessed by analysing 3–4 plots (Table S2; Figure 4). In contrast, near-natural forests (one forest site, approx. 2×5 km) harbour a larger number of rare and unique species even over a smaller range.

Although all randomised species accumulation curves reached saturation with our sampling effort, ICE and Chao 2 richness estimators approach the plateau earlier than first-order Jackknife or Bootstrap (Figure 3). Our study agree with earlier results obtained for wood-inhabiting fungi [15] and suggests that species richness estimators sensitive to singletons and doubletons, like Chao 2 [41] are promising for the analysis of communities with uneven structure found in heterogenic habitats, like yeasts in a soil environment. In summary, our results suggest that the culture-based approach to estimate the diversity of soil yeasts could be optimised by using 50 mixed soil samples plated in triplicates on a single nutrient-rich medium. Still it must be kept in mind that the number of soil samples to be used as replicates from our estimations might only be applicable to a beech forest located in central Europe and further experiments are needed to evaluate the sampling effort in other soil biotopes.

### Implications for diversity assessments of yeasts

Molecular studies on yeast diversity have mainly replaced physiological assimilation tests to identify species as commonly used assimilation tests are not necessarily able to distinguish between closely related species. Additionally, the application of molecular markers has also vastly increased the species numbers in this informal group of fungi [59]–[61]. Numerous yeast genera were identified containing closely related and morphological cryptic species that seem to have evolved via reproductive isolation [62]–[64]. The occurrence of cryptic species will also affect biodiversity assessments in a given habitat and therefore studies based only on morphology might underestimate diversity [65]. Indeed, several authors have suggested that next to the application of different media to culture rare species, the exact identification of cryptic diversity by molecular markers is an important factor that will counter underestimation of yeast diversity found in a given area [66]–[67].

In this study, the used plating strategy resulted in an average of 4.7 colonies per plate, including plates with no yeast colonies. This enabled a reliable differentiation of colonies and adequate isolation of representative strains. Consequently, two new species, described as *Clavispora reshetovae* and *Barnettozyma vustinii*, were found during this survey [68]–[69]. Out of 18 isolated different phylogenetic species in our study, four belong to the anamorphic yeasts genus *Cryptococcus*. However, only *Cryptococcus ramirezgomezianus* and *Cr. musci* (Trichosporonales) can be interpreted as real cryptic species, as they are difficult to distinguish morphologically [20]. Whether they occur in the same or a similar ecological niche, as could be assumed from their similar assimilation profile [20], still needs further studies. The species pair *Cryptococcus terricola* - *Cr. gastricus* can pose only a minor problem in identification. Although both species belong to the Filobasidiales they are not closely related to each other and exhibit distinct physiological profiles, e.g. assimilation of nitrogen sources [19]. Similarly, *Trichosporon dulcitum* and *T. porosum* differ phenotypically as they belong to two different clades within the order Trichosporonales, which were suggested to be reclassified into distinct anamorphic genera [20], [70]. Based on our results we propose that cryptic species pose no real problem for morpho-physiological biodiversity assessments in temperate forest soils, as we never observed closely related species in the same sample. Nevertheless, this does not eliminate the problem of mislabelling soil-borne yeasts by inappropriate identification tools.

### Conclusion

Proper identification of soil fungi using molecular markers is important for diversity estimations and provides a solid background for reliable comparison of results achieved in different surveys. Our study demonstrated that conventional culture-based experiments can be successfully optimised by using mixed soil samples and a single nutrient-rich medium in order to reduce sampling effort in a given substrate or biotope. Additionally, our results demonstrate the usefulness of quantitative estimations to calculate optimal sampling effort in biodiversity- and monitoring-orientated studies. All species richness estimations performed in this study were based on solid species identification, which should be considered in future high throughput analyses as well. The dissimilarities in the yeast community composition observed at the two different sampling dates might hint to seasonal variation in species occurrence and this observation should be addressed in additional studies with more time points.

## Supporting Information

Figure S1 **Yeast quantity (CFU/g) observed after different pre-cultivation soil treatments.** Whiskers correspond to confidential interval and asterisks to outliers.
(EPS)

Table S1 **Average frequency of occurrence of yeasts in samples after different pre-cultivation treatments (soils collected in September 2007, see** **Figure 1** **) and in relation to the two different management types (all mixed samples, see** **Figure 1** **).**
(DOC)

Table S2 **Species-plot relationship: the number of samples allocated for the analysed combination of plots and the species richness values (Mean and standard deviation).**
(DOC)
