# Supplementary material for: Species Accumulation Curves and Incidence-Based Species Richness Estimators to Appraise the Diversity of Cultivable Yeasts from Beech Forest Soils
Source: PLoS One. 2011 Aug 12;6(8):e23671. doi: 10.1371/journal.pone.0023671 (PMC3155558; doi:10.1371/journal.pone.0023671)
Supplement: Table S1 — Average frequency of occurrence of yeasts in samples after different pre-cultivation treatments (soils collected in September 2007, see Figure 1 ) and in relation to the two different management types (all mixed samples, see Figure 1 ). (DOC) [file pone.0023671.s002.doc]

Table S1. Frequency of occurrence of yeasts in samples after different pre-cultivation treatments (soils collected in September 2007, see Figure 1) and in relation to the two different management types (all mixed samples, see Figure  1).

| Effects: | Pre-cultivation treatments | | Forest management | |
| --- | --- | --- | --- | --- |
| Species | Individual | Mixed | Natural | Managed |
| *Aureobasidium pullulans* | 0.003 | 0.003 | n.o. | 0.004 |
| *Barnettozyma pratensis* | n.o. | n.o. | 0.015 | n.o. |
| *B. vustinii* | n.o. | n.o. | n.o. | >0.001 |
| *Candida kruisii* | n.o. | >0.001 | n.o. | 0.011 |
| *C. sake* | >0.001 | 0.005 | 0.004 | 0.042 |
| *C. vartiovaarae* | n.o. | n.o. | 0.017 | 0.011 |
| *Cryptococcus gastricus* | 0.086 | 0.052 | 0.047 | n.o. |
| *Cr. musci* | 0.001 | 0.011 | n.o. | 0.014 |
| *Cr. ramirezgomezianus* | 0.003 | 0.017 | n.o. | 0.019 |
| *Cr. terricola* | 0.033 | 0.018 | 0.005 | 0.242 |
| *Debaryomyces hansenii* | 0.011 | 0.003 | 0.011 | 0.014 |
| *Guechomyces pullulans* | >0.001 | 0.003 | 0.017 | 0.011 |
| *Kazachstania piceae* | >0.001 | 0.007 | 0.061 | 0.125 |
| *Lindnera misumaensis* | n.o. | n.o. | n.o. | 0.016 |
| *Rhodotorula glutinis* | 0.006 | 0.003 | 0.003 | n.o. |
| *Trichosporon dulcitum* | 0.553 | 0.528 | 0.558 | 0.342 |
| *Trichosporon* cf. *laibachii* | >0.001 | 0.046 | 0.051 | 0.006 |
| *Trichosporon porosum* | 0.225 | 0.082 | 0.050 | 0.018 |
