# Supplementary material for: Species Accumulation Curves and Incidence-Based Species Richness Estimators to Appraise the Diversity of Cultivable Yeasts from Beech Forest Soils
Source: PLoS One. 2011 Aug 12;6(8):e23671. doi: 10.1371/journal.pone.0023671 (PMC3155558; doi:10.1371/journal.pone.0023671)
Supplement: Table S2 — Species-plot relationship: the number of samples allocated for the analysed combination of plots and the species richness values (Mean and standard deviation). (DOC) [file pone.0023671.s003.doc]

Table S2. Species-plot relationship: the number of samples allocated for the analysed combination of plots and the species richness values (Mean and standard deviation).

|  | Number of samples | | | Observed species richness, N | | Estimated species richness, N | |
| --- | --- | --- | --- | --- | --- | --- | --- |
| Number of plots | Sept-2007 | Apr-2008 | Total | Natural forests | Managed forests | Natural forests | Managed forests |
| 2 | 30*1 Plot=30 | 5*1 Plot=5 | 35 | 8.2 | 9.6 | 9.83±1.8 | 11.97±2.1 |
| 3 | 30*1 Plot=30 | 5*2 Plots=10 | 40 | 9.2 | 11.0 | 10.43±1.0 | 14.39±4.0 |
| 4 | 30*1 Plot=30 | 5*3 Plots=15 | 45 | 10.0 | 11.4 | 11.50±1.3 | 16.85±4.5 |
| 4 | 30*2 Plots=60 | 2*5 Plots=10 | 70 | 12.0 | 11.6 | 13.19±1.1 | 14.12±2.0 |
| 5 | 30*1 Plot=30 | 4*5 Plots=20 | 50 | n.a. | 11.8 | n.a. | 16.14±4.5 |
| 5 | 30*2 Plots=60 | 3*5 Plots=15 | 75 | 14.0 | 12.0 | 15.75±1.0 | 14.56±2.1 |

Abbreviation used: n.a., not applicable
